# Compliant immune response of silk-based biomaterials broadens application in wound treatment

**DOI:** 10.3389/fphar.2025.1548837

**Published:** 2025-02-12

**Authors:** Zhiqiang Tian, Hong Chen, Ping Zhao

**Affiliations:** ^1^ Biological Science Research Center, Integrative Science Center of Germplasm Creation in Western China (CHONGQING) Science City, Southwest University, Chongqing, China; ^2^ Department of Orthopedics, 903 Hospital of Joint Logistic Support Force of The People’s Liberation Army, Hangzhou, China

**Keywords:** biopharmaceuticals, nanomedicine, sericin, silk fibroin (SF), wound treatment

## Abstract

The unique properties of sericin and silk fibroin (SF) favor their widespread application in biopharmaceuticals, particularly in wound treatment and bone repair. The immune response directly influences wound healing cycle, and the extensive immunomodulatory functions of silk-based nanoparticles and hydrogels have attracted wide attention. However, different silk-processing methods may trigger intense immune system resistance after implantation into the body. In this review, we elaborate on the inflammation and immune responses caused by the implantation of sericin and SF and also explore their anti-inflammatory properties and immune regulatory functions. More importantly, we describe the latest research progress in enhancing the immunotherapeutic and anti-inflammatory effects of composite materials prepared from silk from a mechanistic perspective. This review will provide a useful reference for using the correct processes to exploit silk-based biomaterials in different wound treatments.

## 1 Introduction

Silk is a natural fiber with a long history. It is formed by the solidification of the silk liquid secreted by the mature silkworm during the cocoon phase. Silk is mainly composed of hydrophobic silk fibroin (SF), which forms the core fiber, and hydrophilic sericin, a globular protein that binds to SF (Johari et al., 2022; Silva et al., 2022). SF is rich in glycine, alanine and serine, while sericin mainly contains serine and threonine (Silva et al., 2022; Takahashi et al., 1999).

Initially, sericin was often discarded in large quantities as textile waste, resulting in environmental pollution and wastage of natural resources (Aramwit et al., 2012). With the rapid development of biomaterials, the use of sericin in medicines has received increasing attention. Sericin possesses outstanding antioxidant, anti-inflammatory, antibacterial, antiviral, and biological characteristics that promote tissue regeneration, making it important in curing diseases such as hypertension, cancer, and diabetes (Hu et al., 2022). In addition, sericin biomaterials exhibit good biocompatibility and low immunogenicity, and have been engineered into various biomaterials, including films, hydrogels, scaffolds, fiber pads, particles, coatings, conduits, and nanoparticles for tissue repair, regeneration, and disease cure (Qi et al., 2018; Shah et al., 2019; Li et al., 2020; Wang et al., 2021; Xu et al., 2022; Deng Y. et al., 2022; Ghensi et al., 2019; Fu et al., 2022). Over the past decade, sericin-based biomaterials have developed rapidly in drug-delivery and tissue engineering.

SF has superior biocompatibility, marked mechanical properties, controlled biodegradation rates, and ideal cellular-SF interactions (Gholipourmalekabadi et al., 2020; Wani et al., 2022; Wang et al., 2008). The application of silk in the textile industry has a long history and the preparation technology for silk is very mature; therefore, the source of SF is rich (Huang W. et al., 2018). These characteristics have made SF emerge among biomaterial applications in recent years. SF can be transformed into scaffolds, hydrogels, films, microspheres, and nanoparticles alone or in combination with other materials for tissue engineering such as nerves, bone and muscles, and can also be used in drug delivery systems for skin wound treatment, tumor immunotherapy, and other medical treatments (Cui et al., 2020; Wang D. et al., 2022; Wenhao et al., 2020; Del Bianco et al., 2022; Cai et al., 2023; Hassan et al., 2024).

The body possesses intricate and precise protective mechanisms. When silk is used as a medical material, the immune system often faces challenges. Silk medical materials require different processing techniques before implantation into the body, which can cause significantly alteration of the protein structure of the original silk, resulting in varying levels of immune system activation. This difference may be due to surface chemistry, protein conformation, and polymer formation with other proteins in the treated silk-based materials (Majumder et al., 2024). This has expanded the application of silk in the medical field and has extended the proinflammatory effects to anti-tumor, antibacterial, and vaccine adjuvant fields, although a low immune response contributes to bone, skin, and vascular repair. In this review, we aimed to provide profound discussion of the activation of a wide range of immune responses *in vivo* using silk-based biomaterials and describe the rigorous and accurate processing methods required for different clinical applications in subsequent research.

## 2 Immune responses induced by silk material in mammals

One of the most important characteristics of biomaterials is their biocompatibility, the initiation of the innate immune cells is the decisive factor in the biocompatibility of biomaterials. This process usually causes an inflammatory response, and the degree of the response resides mainly on the properties of the biomaterial (Ekdahl et al., 2011). In this section, we summarize and discuss the biosafety, immunogenicity and immunomodulatory properties of sericin and SF.

### 2.1 Sericin

#### 2.1.1 Favorable biocompatibility and low immunogenicity

**FIGURE 1:**
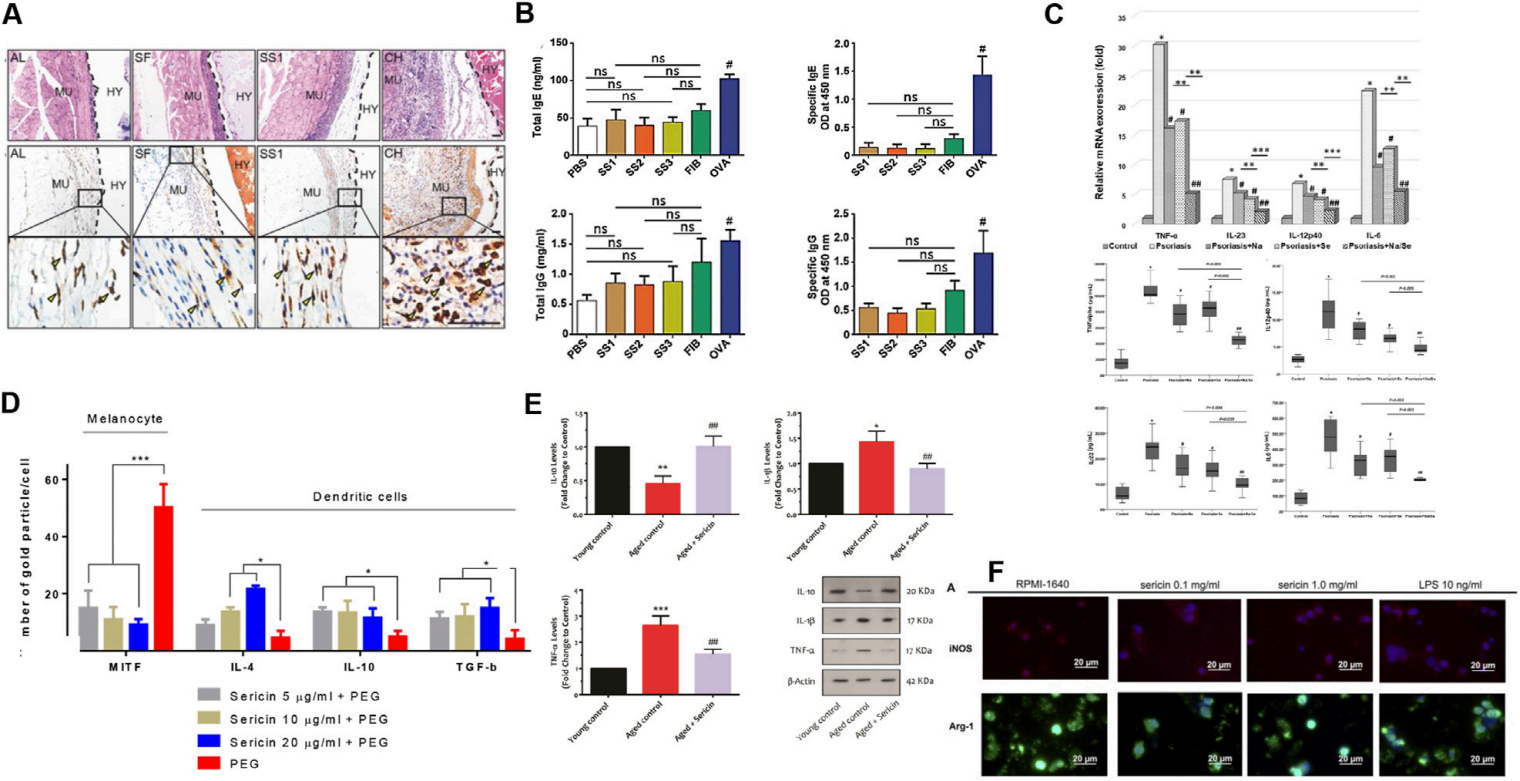
Sericin presents characteristics of good biocompatibility, low immunogenicity, inhibition of inflammatory responses, and good immune regulatory function. **(A)** Hematoxylin and eosin (H&E) staining following 10 days of implantation of AL, SF, SS1 and CH. AL, alginate; SF, silk fibroin; SS, sericin; CH, chitosan; MU, mouse muscles; HY, hydrogel-implanted sites. **(B)** ELISA results measuring total IgE, allergen-specific IgE, IgG (total) and induced by sericin, fibrinogen, PBS, and ovalbumin (OVA). Reprinted with permission from (Jiao et al., 2017). Copyright (2017) Wiley-VCH. **(C)** mRNA expression and cytokine production of proinflammatory factor in hPBMC from the psoriasis patients, who exposure to naringin (20 μg/mL), sericin (100 μg/mL), or sericin/naringin. Reprinted with permission from (Deenonpoe et al., 2019). Copyright (2019) BMC. **(D)** Expression of microphthalmia-associated transcription factor (MITF) and IL-4, IL-10, and TGF-β on melanocytes and DC 48 h after allergy induction treated with PEG or sericin (5, 10 and 20 μg/mL) + PEG. Reprinted with permission from (Aramwit et al., 2018). Copyright (2018) BMC. **(E)** Western blotting results of IL-10, IL-1β, and TNF-α in the young mice, adult mice, and older adult mice + oral sericin treatment groups (250 mg/kg, 21 days). Reprinted with permission from (Seyedaghamiri et al., 2021). Copyright (2021) Springer nature. **(F)** Arg-1 and iNOS immunofluorescence staining of macrophage treated with 0.1 mg/mL, 1 mg/mL LMW-sericin (<10 kDa) and 10 ng/mL lipopolysaccharide (LPS). Reprinted with permission from (Cherng et al., 2022).Copyright (2022) Frontiers.

The biosafety of sericin has long been controversial. Studies have confirmed that sericin has good biocompatibility from the three standpoints of inflammation, allergy, and immunogenicity: (i) sericin only causes low response of inflammatory cells *in vivo* (macrophages and neutrophils) (Jiao et al., 2017; Li et al., 2015) (Figures 1A, B); (ii) neglected allergens (Jiao et al., 2017) and (iii) sericin only causes mild innate and adaptive immune responses (Jiao et al., 2017; Zhang et al., 2006; Zhang Y. et al., 2021; Panilaitis et al., 2003). Furthermore, the addition of sericin to a mixture of chitosan and silver nanoparticles (AgNPs) can reduce their immunogenicity (Nayak et al., 2021). Various forms of materials such as nanoparticles, hydrogels, scaffolds, sponges and films prepared from sericin have also not been found to cause marked immune responses or inflammatory reactions (such as mast cell degranulation) (Hassan et al., 2024; Ampawong and Aramwit, 2016). An important reason for the low immunogenicity of serine is that it is rich in hydrophilic amino acids. Inspired by this, poly-β-homoserineand poly-DL-serine materials can substantially reduce foreign body reactions and are expected to replace polyethylene glycol as an ideal implantable biological material (Zhang et al., 2020; Zhang D. et al., 2021) (Table 1).

**TABLE 1:** Application of sericin in the pharmaceutical field and induced immune responses.

Biomaterial	Bio-medical field	Immune cellular response	Effect	Reference
Nanomicelles	Tumor immunotherapy	Promote T cell recruiting; induce DC maturation	Induce anti-tumor immunity	Guo et al. (2022)
Hydrogels	Cancer chemotherapy	Enhance the phagocytic capacity of liver macrophages and promote the proliferation of splenic lymphocytes	Alleviate chemotherapy-induced immunosuppression	Xu et al. (2021)
Microparticles	Psoriasis	Increase the level of TNF-α secreted by LPS-induced human peripheral blood mononuclear cells (hPBMC)	Treat middle-stage psoriasis	Chlapanidas et al. (2014)
Scaffolds	Periodontitis	Downregulate the MMP-9 and MMP-3, upregulate the IL-10 in LPS-stimulated macrophages	Stable anti-inflammatory effect on periodontal disease treatment	Chachlioutaki et al. (2022)
Scaffolds	Chronic nerve compression	Downregulate TNF-α and IL-1β mRNA levels in macrophages	Achieve significant nerve functional recovery in a preclinical CNC animal mode	Zhang et al. (2017)
Hydrogels	Wound repair	Reduce inflammation and TNF-α secretion by macrophages	Promote wound healing	Jiang et al. (2021a)
Nanoparticles	Carrageenan-induced paw edema	Significantly decrease the infiltration of polymorphonuclear cells	Inhibit inflammation induced by carrageenan	Khampieng et al. (2015)
Nanocarriers	Ulcerative colitis	Reduce the infiltration of inflammatory cells in the liver and kidneys	Relieve symptoms of DSS induced UC	Wang et al. (2022b)
Hydrogels	Diabetic wounds	Reduce the infiltration of inflammatory cells at the wound site	Promote the healing of diabetic wounds	El-Samad et al. (2022)
Nanospheres	Ulcerative colitis	Inhibite the LPS-induced inflammatory response of the macrophage cells	Achieve effective therapeutic effects on ulcerative colitis	Xu et al. (2022)
Hydrogels	Ulcerative colitis	Inhibit IL-6 and IL-12 secreted by macrophages	Alleviate UC via wound healing, inhibit inflammation, and inhibit oxidation pathway	Ma et al. (2019)

#### 2.1.2 Anti-inflammatory properties

Inflammation is the body’s defense response to injury or infection and involves a variety of cellular and molecular mechanisms. In the process of tissue healing, inflammatory cells such as macrophages and neutrophils are first recruited to the injury site and release pro-inflammatory factors such as interleukin-1 beta (IL-1β), IL-6, tumor necrosis factor α (TNF-α), etc. These factors promote vascular dilation and increased permeability, attracting more immune cells to participate in the inflammatory response. It also activates the degradation and remodeling of extracellular matrix. Subsequently, anti-inflammatory factors such as IL-4 and IL-10 begin to play a role, inhibiting the production of pro-inflammatory factors, reducing the activity of inflammatory cells, and promoting tissue repair and regeneration (Eming et al., 2017). The balance of inflammation and inflammatory factors is crucial for tissue healing. Excessive inflammatory response may lead to increased tissue damage, while insufficient anti-inflammatory factors may delay the healing process.

Sericin preparations have found application in skin repair, blood sugar reduction, and treatment of acute myocardial infarction (Wang D. et al., 2022; Aramwit et al., 2013; Tuentam et al., 2022; Song et al., 2016) (Figure 1C). There are three main mechanisms through which sericin inhibits the inflammatory response: (i) inhibits infiltration and proliferation of inflammatory cells (Zhaorigetu et al., 2003; Chlapanidas et al., 2013); (ii) inhibits expression of IL-1β, IL-6, IL-23, etc (Song et al., 2016; Chlapanidas et al., 2013; Deenonpoe et al., 2019; Kumar J. P. et al., 2018; Dong et al., 2020; Farajdokht et al., 2021); and (iii) increases the expression of IL-4 andIL-10, etc (Seyedaghamiri et al., 2021; Aramwit et al., 2018) (Figures 1D, E). Notably, Sun et al. revealed the mechanism of sericin inhibiting lipopolysaccharide (LPS) induced inflammation by multi omics integration: (i) sericin inhibits LPS-activated PRRs, Toll-like receptors and NOD-like receptors pathways; (ii) sericin significantly downregulates the expression of the *MyD88* and *NOD1*; (iii) sericin decreases the expression of IL-1β, IL-6, INOS, etc (Sun et al., 2022). Interestingly, I-sericin is induced by γ-irradiation of sericin, and exhibits potent anti-inflammatory activities as the parent molecule, including reduction of oxidative stress-induced inflammatory cytokines cyclooxygenase-2 (COX-2), inducible nitric oxide synthase (iNOS), TNF-α, IL-1β and alleviation of LPS-induced inflammation (Choi et al., 2023; Song et al., 2020).

#### 2.1.3 Good immune regulatory function

Sericin reportedly modulates epidermal immune responses in patients with psoriasis by reducing cytokine production by Th17 cells, upregulating galectin-3 (Lgals3) and down-regulating sphingosine-1-phospholyase 1 (Sgpl1) (Rujimongkon et al., 2021). As recently shown, low molecular weight (LMW) sericin (<10 kDa) enhances immune regulation *in vitro*: LMW-sericin (0.1 mg/mL) can upregulate the expression of CXCL9, IL-12A, BMP-7, and IL-10 in macrophages; balance Th1 and Th2 levels; and induce M2 polarization of macrophages. Sericin regulates macrophage proliferation to achieve immune regulation (Cherng et al., 2022) (Figure 1F). In addition, I-sericin has immune-enhancing effects, manifested as a significant increase in lymphocyte proliferation and activation of NK cells (Song et al., 2020). In addition, biosynthetic sericin 1-like protein can induce tolerant dendritic cells (DCs), which have excellent immunomodulatory capabilities. The purity of sericin 1- like protein is positively correlated with the anti-inflammatory effect of sericin; therefore, it is expected to be developed as an immune modulator (Song et al., 2020; Ritprajak et al., 2021).

In summary, the good biocompatibility and low immunogenicity of sericin are now widely recognized, making it a new avenue for drug delivery and tissue engineering (Wang C. et al., 2022; Sapru et al., 2021). Moreover, the anti-inflammatory properties and good immune regulatory functions of sericin will greatly expand its clinical applications.

### 2.2 Silk fibroin (SF)

SF exhibits good biocompatibility and low immunogenicity; therefore, it is favored for application in biological materials. Many studies have explored inflammatory processes *in vitro* or *in vivo* of SF-based biomaterials in the form of hydrogels, scaffolds, films, and nanoparticles (Table 2).

**TABLE 2:** Application of SF in the pharmaceutical field and induced immune response.

Biomaterial	Bio-medical field	Immune cellular response	Effect	Reference
Nanoreactors	Cancer	Initiate M1 activation; therapy-triggered ICD	Beneficial for systemic tumor clearance	Yu et al. (2023)
Nanomotors	Cancer	Mature dendritic cells, enhance immune cell infiltration, polarize macrophages from M2 to M1, and inhibit Tregs	Causing changes in immunosuppressive TME and activating tumor suppressive immunity	Zhang et al. (2022a)
Nanocomposites	Cancer	Polarize macrophages towards M1, alter immunosuppressive TME	Promote immunotherapy for PD1/PD-L1 checkpoint	Tan et al. (2020)
Nanomotors	Cancer	Reduce the percentage of immunosuppressive Treg cells, activate and recruit tumor-infiltrating lymphocytes	Inhibit the proliferation and growth of primary and metastatic tumor cells	Cao et al. (2022)
Nanofibrous mats	Transcutaneous immunization	Induce effective Th1 and Th2 cellular and humoral immune response	Activation response to OVA	Yang et al. (2020)
Nanofibrous patches	Transcutaneous immunization	Promote the infiltration of T cells	Promote the apoptosis of tumor cells	Hong et al. (2021)
Hydrogels	Diabetes	Promote anti-inflammatory M2 macrophage polarization	Locally regulate the inflammatory response *in vivo*	Kumar et al. (2018b)
Microneedles	Vaccine	Increase B cell responses	Greatly enhanced the humoral immune response of subunit vaccines	Boopathy et al. (2019)
Microneedles	Vaccine	Generate stronger antigen-specific cellular immune responses	Improve protection against lethal influenza challenge in mice	Stinson et al. (2021)
Microneedles	Vaccine	Promote the proliferation of antigen-specific T cells and increase the level of antigen-specific CD8 T cells	Generate stronger cellular and humoral immunity than the initial vaccine	DeMuth et al. (2014)
Nano-adjuvants	Vaccine	Trigger Th1 and Th2 immune responses	Efficient protect to bladder and kidneys	Hasanzadeh et al. (2020)
Nano-adjuvants	Vaccine	Promote the proliferation and differentiation of CD4 TRM cells	Enhance the local immunity of the stomach	Xu et al. (2019)
Hydrogels	Vaccine	Promote the expansion of CD4^+^TRM cell distribution within the gastric epithelium	Enhanced immune response against *Helicobacter felis*	Hu et al. (2020)
Nanoparticles	Immunotherapeutic agents	Enhance the capacity of macrophages to secrete immune cytokines	Notably improve CpG ODN delivery	Zhang et al. (2019)
Hydrogels	Rheumatoid arthritis	Reduce the capacity of THP-1 cells differentiated with Phorbol 12-myristate 13-acetate (PMA) and stimulated with LPS to secrete immune cytokines	Improve rheumatoid arthritis more effectively	Oliveira et al. (2020)
Hydrogels	Skin wounds	Promote M2 macrophage polarization	Accelerate wound healing	Chouhan et al. (2018), Pang et al. (2021), Mei et al. (2022), Qian et al. (2022b), Chen et al. (2023a)
Nanoparticles	Bone regeneration and repair	Promote M2 macrophage polarization	Promote osteoporotic fracture repair	Wang et al. (2022a)
Scaffolds	Bone regeneration and repair	Promote M2 macrophage polarization	Enhance bone regeneration	Xiang et al. (2021), Patel et al. (2022)
Nanoparticles	Ulcerative colitis	Promote M2 macrophage polarization	Alleviate immune response, retard progression and treat UC	Liu et al. (2022)
Nanoparticles	Ulcerative colitis	Increase the CD8 T and B cells, promote M2 macrophage polarization	Regulating innate immune response and enhancing the therapeutic effect of acute colitis	Du et al. (2022)
Nanoparticles	Ulcerative colitis	Promote M2 macrophage polarization	Substantially relieve UC symptoms	Ma et al. (2022)
Nanoparticles	Ulcerative colitis	Promote the secretion of proinflammatory cytokine in macrophages	Significant relief of symptoms of UC disease	Gou et al. (2019)
Nano-micro fibrous woven scaffolds	Tendon tissue engineering	Regulating macrophage polarization towards M2	Notably facilitated Achilles tendon regeneration	Cai et al. (2023)
Scaffolds	Tendon adhesion	Promote M2 polarization of macrophages	Greatly mitigate tendon adhesion	Dong et al. (2021)
Engineering meshes	Pelvic organ prolapse	Promote M2 polarization of macrophages	Enhance tissue repair	Wu et al. (2022)
Hydrogels	Skin wounds	Reduce inflammatory cells	Promote skin appendage formation	Yin et al. (2022)
Scaffolds	Spinal cord injury	Reduce the macrophage/microglia (CD68 positive cells)	Facilitate regeneration of injured spinal cord	Li et al. (2016b)

#### 2.2.1 SF hydrogel induce only a mild inflammatory response

**FIGURE 2:**
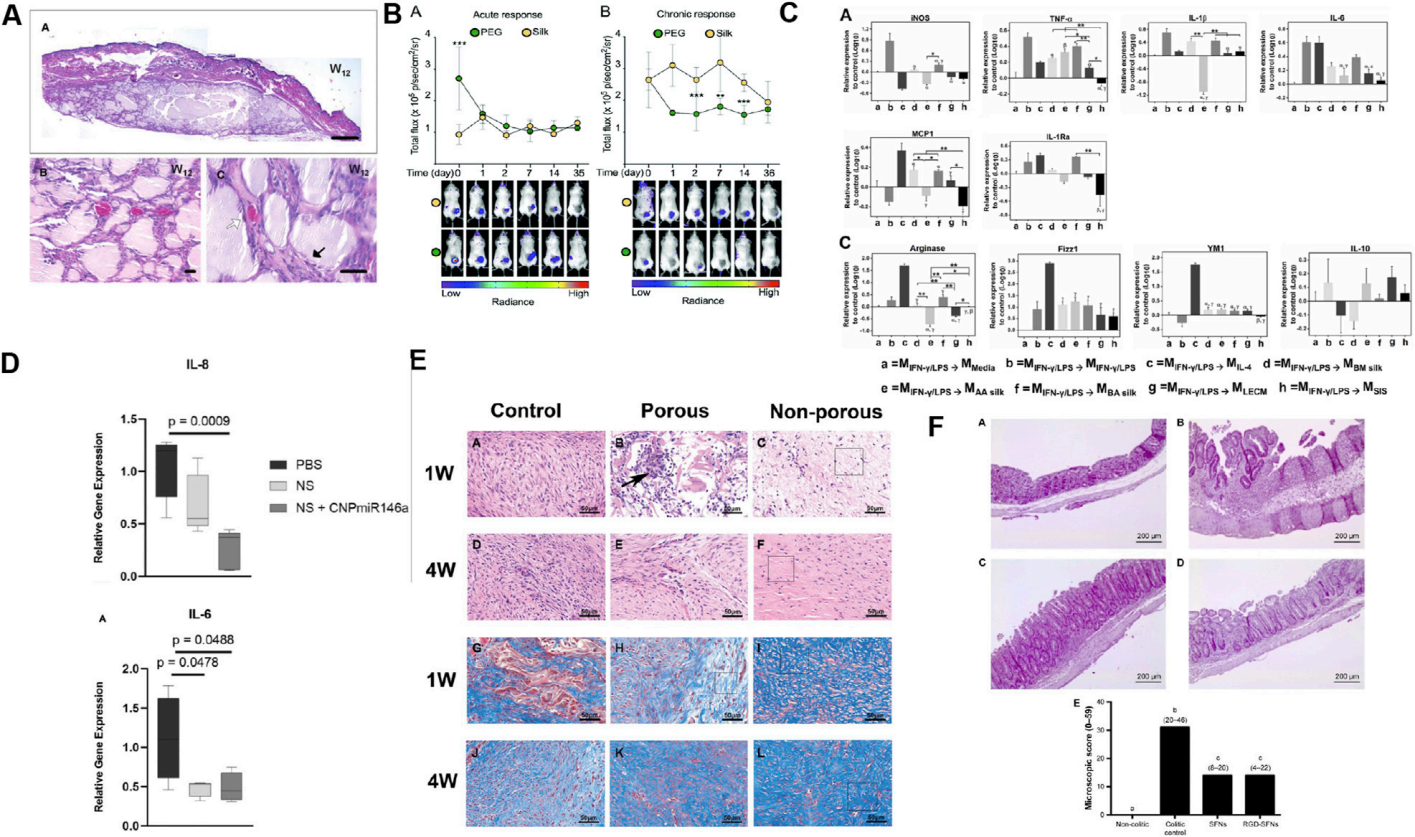
Biomaterials constructed with SF causes a modest inflammatory response and has low immunogenicity and anti-inflammatory properties. **(A)** The H&E staining of skin tissue from the backs of mice subcutaneously transplanted with SF after 12 weeks. Reprinted with permission from (Etienne et al., 2009). Copyright (2009) Wiley-VCH. **(B)** Non-invasive imaging of the acute and chronic inflammatory response towards implanted SF and PEG hydrogels. Reprinted with permission from (Gorenkova et al., 2021). Copyright (2021) the ROYAL SOCIETY of CHEMISTRY. **(C)** Relative expression of proinflammatory and pro-remodeling genes after exposure to degradation products of silk (*Bombyx mori* (BM) silk; *Antheraea assamensi* (AA) silk, and BA silk and ECM (liver ECM (LECM) and small intestinal submucosa ECM (SIS)) bioscaffolds on proinflammatory activated macrophages. Reprinted with permission from (Janani et al., 2022). Copyright (2022) Elsevier. **(D)** H&E staining of colon in non-colitic, colitic control, SFNs and RGD-SFNs group. Reprinted with permission from (Rodriguez-Nogales et al., 2016) Copyright (2016) Dovepress. **(E)** Proinflammatory gene expression of diabetic wounds treated with PBS, nanosilk (made from SF), and nanosilk + cerium oxide nanoparticle-microRNA146a (CNP-miR146a) after 7 days. Reprinted with permission from (Niemiec et al., 2020). Copyright (2020) Frontiers. **(F)** H&E and Masson trichrome staining were performed on the control group, porous SF membrane group, and non porous SF membrane group at 1 and 4 weeks postoperatively. Reprinted with permission from (Yao et al., 2019). Copyright (2019) Wiley-VCH.

SF can achieve solution-gel transition by altering the pH, temperature, and solvation state, or by increasing biopolymer dynamics (Matsumoto et al., 2006). Studies on the biocompatibility and low immunogenicity of hydrogels are often based on the histological evaluation of tissue reactions, with the main focus of observing changes in inflammatory markers, such as neutrophils, eosinophils, and macrophages, in the SF hydrogel and the surrounding tissues (Fu et al., 2022; Etienne et al., 2009; Maity et al., 2022) (Figure 2A). However, these methods are not sufficiently precise to determine the biocompatibility and low immunogenicity of SF hydrogels. Therefore, advanced equipment and technical means are required to explore the inflammatory reaction caused by SF hydrogels in detail. Recently, using noninvasive bioluminescence imaging, Gorenkova et al. demonstrated that SF hydrogels elicited an acute but mild local inflammatory response *in vivo*, which elicited an innate immune response similar to that elicited by polyethylene glycol (PEG) hydrogels (Gorenkova et al., 2021) (Figure 2B). The Forster/fluorescence resonance energy transfer (FRET)-based sensor experiment developed by Kambe et al. first exposed the initial immune response of SF hydrogels. SF hydrogels are surrounded or invaded by matrix metalloproteinases (MMP) within 24 h after implantation and undergo biodegradation within 3 h after implantation, which may favor immune cells (macrophages, foreign body giant cells) to achieve major degradation of the hydrogel over a period of weeks (Kambe and Yamaoka, 2021; Janani et al., 2022) (Figure 2C).

#### 2.2.2 SF scaffolds have low immunogenicity

The surface morphology, physical structure, and chemical structure of the scaffold play a decisive role in the reaction with immune cells, and these features also regulate macrophage polarization at the host tissue implant interface (Antmen et al., 2021). The SF scaffold reportedly has good biocompatibility and low immunogenicity through histological and immunofluorescence staining analysis (Fu et al., 2022; Wang et al., 2008; Guan et al., 2013; Yang et al., 2021; Ge et al., 2012; Suzuki et al., 2019; Singh et al., 2018). Recent studies have revealed the effects of SF scaffolds on macrophages. SF scaffold implants can inhibit classical activated macrophages (M1) and stimulate alternatively activated macrophages (M2) to regulate local inflammatory responses (Janani et al., 2022; Yang et al., 2021). Mast cells, an important type of immune cells, can be activated by biomaterials, which can trigger inflammation by releasing histamine, cytokines and other mediators, promote recruitment and activation of macrophages and other immune cells, and affect the immune microenvironment at the transplant site of biomaterials. Garg K et al. found that SF scaffolds were not conducive to mast cell adhesion and proliferation, suggesting that they were largely immune inert (Garg et al., 2011). The origin, protein conformation, amino acid sequence, fiber thickness, and porosity of SF play important roles in determining the macrophage phenotype, monocyte responsiveness, and T-cell activity (Janani et al., 2022; Yang et al., 2021; Bhattacharjee et al., 2013). Therefore, it is necessary to consider factors that affect the immune response when designing SF scaffolds.

#### 2.2.3 SF nanoparticles/nanofilaments have anti-inflammatory properties and low immunogenicity

Evaluation of the innate and adaptive immunity of SF nanoparticles (SFNPs) *in vivo* demonstrate low immunogenicity and anti-inflammatory properties (Zhang Y. et al., 2021). SFNPs play an anti-inflammatory role and immunomodulatory properties in intestine of trinitrophenyl sulfonic acid-induced experimental colitis in rats, which is specifically manifested as reducing neutrophil infiltration, decreasing the expression of IL-1β and promoting the expression of IL-10, while functionalization of arginine–glycine–aspartic acid (RGD) peptide can significantly improve its anti-inflammatory properties (Rodriguez-Nogales et al., 2016) (Figure 2D). In addition, a SF nanosilk solution reportedly reduces gene expression of the proinflammatory factor IL-6 accompanied by a tendency to reduce inflammatory cell infiltration early in the healing process (Niemiec et al., 2020) (Figure 2E).

SF films are also biocompatible, have low immunogenicity, and can reduce the infiltration of inflammatory cells. SF films are mainly used in wound healing and tissue repair (Ge et al., 2012; Arthe et al., 2020; Yao et al., 2019) (Figure 2F). In addition, the transparent artificial dura made from SF effectively reduces the expression of IL-1β, IL-6, and TNF-α (Kim et al., 2011).

Notably, peptides produced by SF hydrolysis exhibit anti-inflammatory potential. SF peptide alone inhibits TPA-induced increase in COX-2, IL-6, IL-1β, and TNF-α levels, and significantly enhances the anti-inflammatory activity of Tat-SOD and PEP-1-FK506 binding proteins (Kim et al., 2011; Kim et al., 2012). γ-irradiated SF significantly enhances various aspects of the immune systems by activating NK cells, T-cell proliferation, NO production, and increasing cytokine levels (Byun et al., 2010). The anti-inflammatory potential of the SF hydrolysate and irradiation products suggests that further processing and treatment of SF will be a new strategy to explore its value.

## 3 Strategies to reduce the immunogenicity and foreign body reaction (FBR) of sericin-based and SF-based biomaterials

Compared with the current artificial materials, such as polylactic acid, PEG, etc., the degradation products of sericin and SF are small molecular amino acids, and possess lower inflammatory response and better biocompatibility, while the degradation products of artificial materials such as polylactic acid will produce obvious inflammatory response by reducing the pH value of the environment (Ma et al., 2024). Chemical crosslinking is an important strategy to reduce the immunogenicity of biomaterials. Based on the active groups on various amino acid residues in sericin and SF, it can be chemically modified to meet the needs of different applications. For instance, by introducing methacrylic acid group into the amino acid side chain of SF protein, the water solubility of SF protein can be improved, thus reducing the immunogenicity (Kim et al., 2021). Reducing the antigenic epitopes exposed by material surface modification is another important strategy to reduce the immunogenicity. For example, the introduction of specific bioactive molecules, such as PEG, on the surface of fibroin proteins can reduce the immunogenicity of SF (Wei et al., 2020).

Biomaterials implanted in the body will cause FBR, including local aseptic inflammatory responses, such as inflammatory cell infiltration, including macrophages, lymphocytes, neutrophils, etc. Over time, foreign-body giant cell form and eventually lead to fibrosis (Cai et al., 2024). In order to overcome the FBR, it is necessary to pay attention to the following characteristics when preparing sericin-based and SF-based biomaterials. Firstly, the mechanical properties of the hydrogels should be suitable for the implant site, and the lower stiffness helps to mitigate FBR (Blakney et al., 2012). Secondly, preventing protein adsorption by binding hydrogels with PEG or other novel anti-fouling biomaterials (such as zwitterionic or hydroxyl-rich polypeptides) is another attractive option (Zhou et al., 2024). Another method to mitigate FBR is to use biomimetic materials (such as decellularized extracellular matrix and mucins) mimic the extracellular matrix (ECM) of native tissues (Bhunia et al., 2023; Werlang et al., 2024). In regard to nanoparticles, surface functionalized by different chemical groups can affect the intensity of the FBR (Huang Y. J. et al., 2018; Huang et al., 2015). It is noteworthy that the membrane encapsulation to reduce the FBR of the nanoparticle has become another novel way (Fan et al., 2018).

Finally, the composition and MW of sericin and SF also play a key role in its immunogenicity. SF is a fibrous protein consisting of a heavy chain (H chain) (390 kDa), a light chain (L chain) (26 kDa), and a glycoprotein P25 (30 kDa), which are assembled in a ratio of 6:6:1 (Inoue et al., 2000). Sericin is also a macromolecular protein, with MW ranges from about 10 kDa to over 300 kDa (Zhang, 2002). The MW of them is greatly affected by the extraction conditions. LMW-sericin (below 10 kDa) not only displays good biocompatibility, but also owns good anti-inflammatory ability to regulate macrophage polarization towards the M2 phenotype (Cherng et al., 2022). Therefore, it is another way to reduce the immunogenicity of sericin-based and SF-based biomaterials by optimizing the extraction and purification process to obtain the appropriate MW of sericin and SF.

## 4 Application of sericin biomaterial in medicine

Due to the good biocompatibility, low immunogenicity and outstanding immunomodulatory properties, sericin is highly favored in the biomedical field. Sericin-based biomaterials have shown excellent effects in improving immunotherapy and anti-inflammatory.

### 4.1 Improving immunotherapy

Immunotherapy is mainly a method of treating diseases by artificially enhancing or inhibiting the body’s immune function. It is suitable for treating various diseases, including cancer and autoimmune diseases. Currently, the drug delivery system using sericin as a biological material for immune agents become a very promising method for immunotherapy.

#### 4.1.1 Enhance anti-tumor immunotherapy

Small interfering RNA (siRNA) is essential for the effective inhibition of tumorigenesis, targeting of tumor metastasis, and activation of tumor-associated immune cells via silencing the specific gene (such as p65 and PD-L1) responsible for different cancer hallmarks (Ngamcherdtrakul and Yantasee, 2019). Using an effective delivery system, siRNAs can be selectively targeted to tumor microenvironment (TME), sent to regulatory T cells (Tregs), macrophages, myeloid-derived suppressor cells, and other cells to “silence” immunosuppressive cells, and enhance therapeutic immunotherapy (Deng K. et al., 2022; Qian H. et al., 2022; Li S. Y. et al., 2016; Hossain et al., 2015). Currently, sericin as a biological material has been designed as a delivery system for targeted delivery of siRNA. For example, a system consisting of superparamagnetic iron oxide nanoparticles (SPIONs) modified with sericin can target triple-negative breast cancer, accelerate tumor necrosis, and inhibit tumor proliferative growth (Shirangi et al., 2022). Furthermore, hyaluronic acid/poly-L-lysine-siRNA/albumin-sericin (2:1) nanoparticles can be used as siRNA delivery system for laryngeal cancer treatment (Yalcin et al., 2019). Therefore, it is expected that, in the future, serine will be increasingly used as a biological material to design a drug delivery system to target siRNA to immunosuppressive cells in the TME, which will be a potential new direction for enhancing immunotherapy.

**FIGURE 3:**
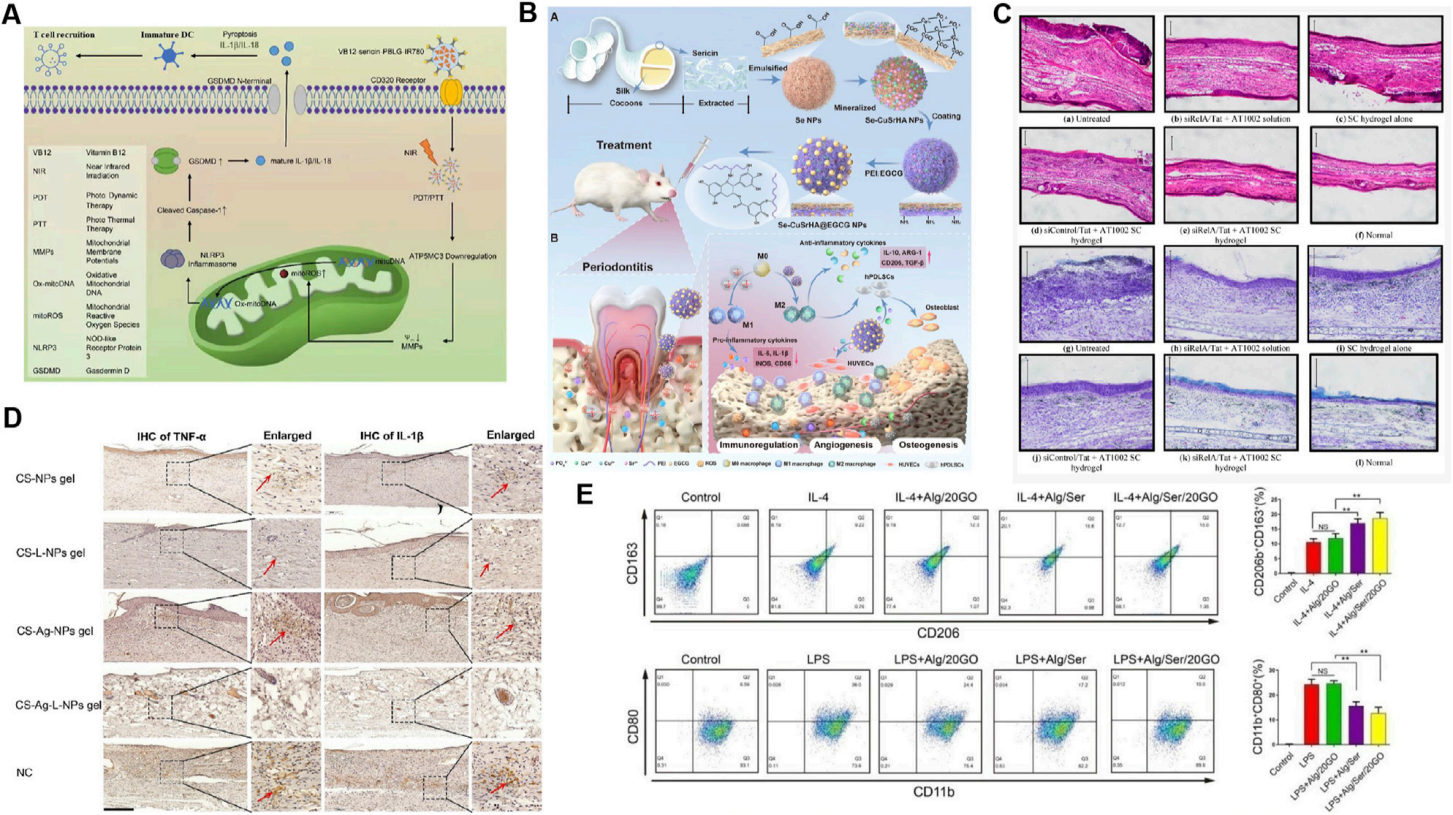
Application of sericin-based biomedical composites in immune regulation. **(A)** The preparation process and mechanism of VB12-sericin-PBLG-IR780 nanomicelles mediated pyroptosis are related to DC maturation, T cell recruitment, and tumor inhibition efficiency. Reprinted with permission from (Guo et al., 2022). Copyright (2022) ACS. **(B)** Treatment of atopic dermatitis in mice with siRelA sericin (SC) hydrogel. Observation of ear swelling (HE staining) and mast cell infiltration (TB staining) in tissue sections through optical microscopy. Reprinted with permission from (Kanazawa et al., 2015). Copyright (2015) MDPI. **(C)** Se-CuSrHA@EGCG Preparation and functional model diagram of nanoplatforms, including clearance of ROS, regulation of immune response, angiogenesis, and bone formation. Reprinted with permission from (Ming et al., 2025). Copyright (2025) Elsevier. **(D)** Chitosan (CS) - NPs gel, CS Lupeol (L) - NPs gel, CS Ag NPs gel and CS Ag L-NPs gel were used to treat skin wounds. The expression of TNF - α and IL-1 β in tissues was detected by immunohistochemistry. Reprinted with permission from (Chu et al., 2023). Copyright (2023) Elsevier. **(E)** Treated with IL-4 and alginate/sericin/graphene oxide (Alg/Ser/GO) hydrogel, the frequency of CD206^+^CD163^+^and CD11b^+^CD80^+^cells was analyzed by flow cytometry. Reprinted with permission from (Jiang et al., 2021a). Copyright (2021) Elsevier.

Photothermal therapy (PTT) and photodynamic therapy (PDT) are two novel cancer treatments. Their anti-tumor efficacy can be improved by inducing non-invasive pyroptosis of cancer cells and stimulating anti-tumor immune responses. For example, recently prepared VB12-Sericin-PBLG-IR780 nanomicelles not only trigger programmed pyroptosis in cancer cells but also activate DC maturation, initiate T-cell recruitment, and play a key role in anti-tumor processes (Guo et al., 2022) (Figure 3A).

In addition, during the treatment for tumors, although the use of chemotherapy drugs inhibits tumor formation and development, it can also have a negative impact on the immune system. Lactoferrin (LF), a common iron-binding glycoprotein, not only has the function of regulating iron metabolism, but also plays a crucial role in antibacterial, antiviral, anti-tumor and immune regulation (Jańczuk et al., 2022). A sericin hydrogel system for the delivery of recombinant human LF (SSH-rhLF) can prolong the bioactivity and bioavailability of rhLF and may have a therapeutic effect on the cyclophosphamide (CTX) induced immunosuppression mice by enhancing the function of liver macrophages, promoting the expression of immunoregulatory factors (IL-2, IL-21, IL-18, and CD-3), and promoting the proliferation of splenic lymphocytes (Xu et al., 2021). This provides a new strategy for enhancing immunity in patients undergoing cancer chemotherapy.

#### 4.1.2 Involvement in immune regulation of autoimmune diseases

Atopic dermatitis (AD) is a common chronic inflammatory disease, for which immunotherapy is an important treatment (Li and Man, 2022). Sericin prepared in emulsion gel and hydrogel forms is very effective in correcting the abnormal immune response caused by AD by loading levocetirizine (an antihistamine), anti-RelA siRNA, and functional peptides (Pal et al., 2019; Kanazawa et al., 2015) (Figure 3B). Intra-articular injection of sericin hydrogel preparations containing anti-RelA siRNA also has great therapeutic potential for the treatment of rheumatoid arthritis (RA) (Kanazawa et al., 2017). Moreover, sericin-based microspheres loaded with racemic flavanone naringenin (a TNF-α blocker) helped to suppress LPS-induced serum TNF-α levels, which mediate immune disorders in psoriasis, and thus are expected to be a new modality for psoriasis immunotherapy (Chlapanidas et al., 2014).

### 4.2 Role in anti-inflammatory activity

Sericin has good adhesion and hydrophilicity, which helps to regulate the mechanical properties of biomaterials, enhance their degradation ability, promote cell adhesion and proliferation, and facilitate the sustained release of anti-inflammatory drugs, thereby enhancing their anti-inflammatory effects. From a mechanistic perspective, the drug delivery system and composite biomaterials involved in sericin play an anti-inflammatory role, mainly by promoting the M2 polarization of macrophages, inhibiting the proliferation and infiltration of inflammatory cells, and regulating the secretion of inflammatory mediators.

#### 4.2.1 Inhibition of inflammation by promoting M2 polarization of macrophages

Macrophages are among the first cells to arrive at and interact with implanted materials and involved in regulating the resolution of inflammation, promoting tissue repair and regeneration. Mature macrophages are polarized into M1 or M2 subtypes. Classically activated M1 induced by IFN-γ, exhibit a proinflammatory phenotype. Activated M2 induced by IL-4 or IL-13 exhibit an anti-inflammatory phenotype (Kumar et al., 2016). During tissue repair, excessive and prolonged activation of proinflammatory M1 macrophages in the early stages of inflammation can lead to increased inflammation, thereby impairing tissue repair and regeneration (Martin and García, 2021; Bessa-Gonçalves et al., 2023). Therefore, accelerating the change in the macrophage from M1 to M2 is an important strategy in both biomaterial development and tissue repair/regeneration.

The polarization response of macrophages to biomaterials is currently being explored in three main approaches: (i) immunofluorescence staining using M1 surface markers (such as chemokine receptors 7, CCR7) and M2 surface markers (such as CD206) and observing results (Wang et al., 2020); (ii) determining cytokine secretion profiles of macrophages, which indirectly reflect macrophage phenotype (Wang et al., 2020; Chachlioutaki et al., 2022); (iii) examining the expression of M1 and M2 genes (Wang et al., 2020; Zhang et al., 2017; Jiang et al., 2021a). Sericin composite biomaterials in the form of scaffolds, films, hydrogels, and other treatments combined with drugs such as ketoprofen (an anti-inflammatory agent), nerve growth factor, and exosomes inhibit inflammatory responses by promoting macrophage M2/M1 conversion, which is exploited for tissue and peripheral nerve repair (Chachlioutaki et al., 2022; Zhang et al., 2017; Jiang et al., 2021a). Recently, the bone immune microenvironment, in which macrophages play a key role in reprogramming of the bone regeneration and immune microenvironment, has attracted increasing attention. Sericin released in an injectable alginate/serin/graphene oxide (Alg/Ser/GO) hydrogel promotes M2 polarization and migration through nuclear factor kappa-B (NF-κB) and mitogen-activated protein kinase (MAPK) signaling, inducing osteogenic differentiation and bone regeneration (Jiang et al., 2021a). Se-CuSrHA@ (epigallocatechin -3- gallate) EGCG (Figure 3C), a sericin-based nanoplatform, increases mRNA level of *Arg-1* and *CD206*, increases the secretion of anti-inflammatory cytokines, inhibit M1 while promoting the polarization of M2, balanced immune homeostasis, and accelerating bone regeneration (Ming et al., 2025). In another study, serine incorporated into a gelatin sponge polarizes M1 macrophages and promotes bone morphogenetic protein 2(BMP-2) secretion to enhance osteogenesis (Jo et al., 2021). Therefore, the factors through which sericin materials determine macrophage phenotypes may be complex and require further exploration.

#### 4.2.2 Inhibition of the infiltration and proliferation of inflammatory cells

Sericin-composite biomaterials can also inhibit inflammatory cell proliferation and infiltration in the treatment of inflammatory diseases. Sericin-loaded alginate nanoparticles significantly reduce polymorphonuclear cell (PMN) infiltration and inhibit carrageenan-induced paw edema (Khampieng et al., 2015). The sericin/proanthocyanidin (PAC) composite reverses histological damage, including inflammatory cell infiltration and goblet cell loss, slowing disease progression and is a promising alternative therapeutic strategy for dextran sulfate sodium (DSS) -induced ulcerative colitis (UC) (Wang C. et al., 2022). In addition, the reduction of inflammatory cells in the surrounding tissues was observed at the initial stages of implantation of the alginate/sericin/graphene oxide (Alg/Ser/GO) hydrogel, which is conducive to bone regeneration (Jiang et al., 2021a). Sericin can promote chondrocyte differentiation and growth by activating the Smad2/3/TGF-β pathway and regulate local proinflammatory responses (Fongsodsri et al., 2024).

#### 4.2.3 Regulate the release of inflammatory factors

In terms of wound treatment, carboxymethyl cellulose/sericin-based hydrogel dressing can downregulate the IL-1β, IL-6, and TNF-α to improve the pro-inflammatory response at the diabetic wound site (El-Samad et al., 2022; Bai et al., 2023). Hydrogels made of fibroin and sericin are excellent at promoting wound healing and reducing inflammation (Zhang D. et al., 2024; Ashraaf et al., 2023). Using sericin as a biological template, CuS@Ser NPs stimulate angiogenesis and inhibited inflammation, thereby facilitating rapid wound healing (Guo et al., 2023).

Another bacterial cellulose wound dressing made from sericin/polyhexamethylene biguanide has a strong ability to promote tissue secretion of IL-4 and TGF- β, thereby achieving a more efficient regulatory ability to promote wound treatment (Napavichayanun et al., 2018). In terms of psoriasis treatment, the combination of naringin and sericin in equal proportions significantly reduced the production IL-6, TNF-α and IL-23 in patients (Deenonpoe et al., 2019). Sericin-based poly (vinyl) alcohol (SS/PVA) hydrogel alleviates the development of psoriasis symptoms by up-regulating nuclear factor erythroid 2 related factor 2 (Nrf2), IL-10, and down-regulating TNF-α and IL-20 (Tuentam et al., 2022). In terms of colitis treatment, sericin composite biomaterials can alleviate UC by reducing the levels of proinflammatory cytokines TNF-α, IL2, IL-6, IL-17, and IL-12 and upregulating the levels of the anti-inflammatory cytokine IL-10 (Xu et al., 2022; Fongsodsri et al., 2024; Ma et al., 2019). The mixture of sericin and curcumin can inhibit carrageenan-induced foot edema in mice by suppressing the release of IL-1 β, promoting the secretion of IL-4 and IL-10 (Ashraaf et al., 2023).

The sericin composite biomaterials loaded with drugs can be used to treat inflammatory diseases by reducing the release of proinflammatory mediators, upregulating anti-inflammatory cytokines and macrophage polarization (Jiang et al., 2021a; Chu et al., 2023) (Figures 3D, E). Sericin base biomaterial is a new and gradually emerging therapeutic strategy for wound treatment.

## 5 Application of SF in medicine

Compared with sericin, SF is more widely used in the field of tissue engineering and regenerative medicine attributed to its better mechanical properties and versatile processing capabilities. SF-based biomaterials have also played an outstanding role in enhancing immunotherapy and anti-inflammatory.

### 5.1 Improving immunotherapy

#### 5.1.1 Enhanced anti-tumor immune response

**FIGURE 4:**
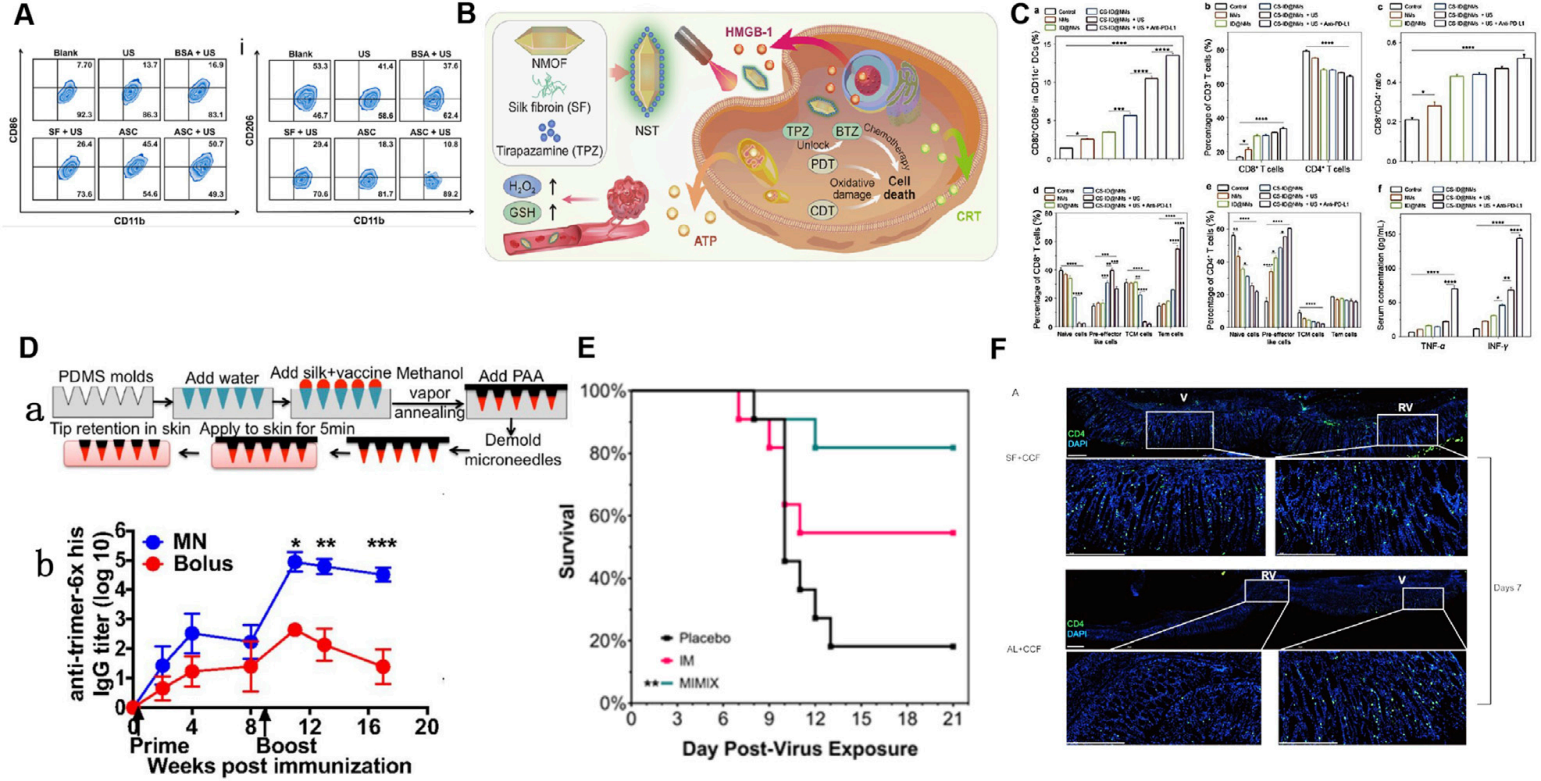
Application of SF-based biomedical composites in anti-tumor immune response and enhancing the immune response to vaccines. **(A)** Flow cytometry analysis showing the frequency of M1 and M2 macrophages after various treatments: ultrasound (US), bovine serum albumin (BSA)+US, SF + US, Au/SF@Cu2-xS nanoreactor (ASC), and ASC + US. Reprinted with permission from (Yu et al., 2022). Copyright (2023) Elsevier. **(B)** Schematic illustration of the synthetic route of NST NPs (metal-organic framework nanosystem (NMOF)+SF + tirapazamine (TPZ)) and the mechanism of synergistic induction of strong immune response by deoxygenation driven chemotherapy in the treatment of tumor specific redox imbalance. Reprinted with permission from (Yu et al., 2022a). **(C)** Impact of different types of oral nanomotors (NMs)-embedded hydrogel plus anti-PD-L1 on DC maturation, the ratio of CD8^+^/CD4^+^ T cells and changes, central memory cells (TCM), effector memory T cells in the mouse spleen and variations in cytokine levels (e.g., TNF-α and IFN-γ) in the mice serum. Reprinted with permission from (Cao et al., 2022). Copyright (2022) Wiley-VCH. **(D)** (a) Schematic of the fabrication and application to skin of SF/poly (acrylic acid) (PAA) composite microneedles. (b) ELISA analyses of anti-trimer-His serum IgG responses over time. Reprinted with permission from (Boopathy et al., 2019). Copyright (2018) PNAS. **(E)** Kaplan–Meier survival curves showing that MIMIX (SF-based microneedle patch) reduced mortality relative to control, whereas bolus vaccination vaccines did not significantly improve survival rates. Reprinted with permission from (Stinson et al., 2021). Copyright (2022) Elsevier. **(F)** Immunofluorescence staining in acute or steady-state conditions: Adding SF to vaccines can induce stronger immune responses and increase the distribution of CD4^+^T cells in the gastric mucosa. Reprinted with permission from (Hu et al., 2020). Copyright (2020) Taylor and Francis.

SF has biocompatibility, controlled-release characteristics, and excellent mechanical and biological properties, such as immunomodulatory and anti-inflammatory properties, making it an excellent choice for the construction of composite biomaterials for tumor immunotherapy. Surface-engineered SF nanocomposites not only exhibit excellent anti-tumor functions but can also be combined with different types of tumor therapies to enhance tumor immunotherapy. The related mechanisms mainly include: (i) reversal of the immunosuppressive TME, including inducing the transformation of tumor-associated macrophages from M2 to M1 and reducing the number of Tregs (Zhang X. et al., 2022; Yu et al., 2022a; Tan et al., 2020; Yu et al., 2023) (Figures 4A, B); (ii) induction of immunogenic cell death (ICD) to stimulate anti-tumor immunity (Zhang X. et al., 2022; Yu et al., 2022a; Tan et al., 2020; Yu et al., 2023; Tkach et al., 2017); and (iii) promotion of the maturation of DCs, the formation of effector T cells and effector memory T cells to improve immune activation and promote immune memory (Zhang X. et al., 2022; Yu et al., 2022a; Tan et al., 2020; Tkach et al., 2017; Cao et al., 2022) (Figures 4A–C).

Transcutaneous immunization (TCI) enhances tumor immunotherapy by delivering antigens to DCs through skin. Compared to traditional oral or injection vaccinations, it has the advantages of excellent immunogenicity, avoidance of the liver first-pass effect, good compliance, safety, high efficiency, non-invasiveness, and ease of use (Karande and Mitragotri, 2010). However, the presence of a cuticular barrier leads to a low transdermal delivery efficiency of TCI, thereby limiting its large-scale clinical application. SF has good biocompatibility, air permeability, and skin affinity, and can mimic the extracellular matrix, making it a good biological material for constructing transdermal drug delivery systems. Composite biomaterials constructed using SF can Stimulate cellular and humoral immune responses and induce systemic anti-tumor response by improving the transdermal properties of transdermal carriers, targeting, and inducing DCs maturation (Yang et al., 2020; Song et al., 2022). According to recent researches, the combination of SF-constructed percutaneous tumor immune materials and immune checkpoint blockers, such as programmed cell death protein 1 monoclonal antibody (aPD-1) which can enhance T-cell responses and mediate preclinical antitumor activity by blocking the binding of PD-1 on T cell with PD-L1 on cancer cells, may be a new strategy for effectively enhancing tumor immunotherapy. The involved mechanisms include: (i) promotion of the infiltration of CD4 and CD8 T cells into the tumor tissue, and (ii) promotion of the expression of IL-12, IFN-γ, and TNF-α (Song et al., 2022; Hong et al., 2021).

It is also of interest that SF has been used as a vaccine carrier for cancer immunotherapy. For example, Lei et al. recently developed an injectable SF microsphere loaded with an antigen and an immune adjuvant. Its macroporous structure is conducive for the recruitment of immune cells and can promote the activation of DCs to forms a favorable immune microenvironment. In turn, strong humoral and cellular immunity is induced. In addition, an enhanced vaccine modified by adsorbing antigens on SF microspheres effectively inhibits tumor growth by improving the cytotoxic T lymphocyte (CTL) response (Lei et al., 2022), properties that encourage a new approach for the future development of tumor immunotherapy vaccines.

#### 5.1.2 Enhanced immune response to vaccines

Antigen delivery dynamics can influence the immune response to vaccines. For example, vaccine antigens can induce sustained humoral immunity after they are delivered to the lymph nodes to trigger naïve B cell response, whereas traditionally injected immunization can rapidly eliminate antigens, which is not conducive to the establishment of humoral and cellular immunity. Improving the immunogenicity of vaccine antigens and maintaining their slow release are new strategies for enhancing vaccine efficacy. The ability of the SF matrix to enclose and release intact and bioactive immunologically active materials has attracted much attention in the construction of slow-release novel vaccines (Reeves et al., 2015; Guziewicz et al., 2011; Kumar M. et al., 2018) (Figure 4D). The microneedle patch combined with the SF matrix can achieve the continuous release of vaccine antigens, enhance the immunogenicity of the vaccine, and thereby significantly enhance the degree, duration, and breadth of the humoral and cellular immune responses caused by the vaccine. Therefore, this represents a promising vaccine delivery strategy at present (Boopathy et al., 2019; Stinson et al., 2021; DeMuth et al., 2014) (Figure 4E).

SFNPs can enhance antigen target delivery, immunogenicity, and stability and can release antigens slowly and continuously, making them a promising new vaccine preparation. SFNPs was used as nanoadjuvants to deliver recombinant hepatitis B surface antigen (HBsAg) and FimH-IutA antigen, with the resulting vaccine significantly increasing the content of specific antibody IgG and promoting humoral and cellular immune responses (Rezaei et al., 2021; Hasanzadeh et al., 2020). Similarly, SF, which presents the advantages of sustained release, absorption, and *in situ* gelation in various tissues, has been used as a mucosal vaccine carrier. This vaccine carrier not only alleviates gastric injury but also leads to significant infiltration and generation of CD4 tissue-resident memory T (TRM) cellsin gastric epithelial tissues, which are the key mediators of anti-infection immunity in various tissues and have recently been shown to boost local stomach immunity (Schenkel et al., 2014; Xu et al., 2019; Hu et al., 2020) (Figure 4F).

#### 5.1.3 Enhanced efficacy of immunotherapy drugs

CpG oligodeoxynucleotides (CpG ODNs) are short single-stranded synthetic DNA molecules which are designed to mimic bacterial DNA. These molecules are recognized by Toll-like receptor 9 (TLR9), which is expressed in certain immune cells such as myeloid cells, thus possess potent immune-stimulatory properties (Hekmatshoar et al., 2019). SFNPs have been used as effective carriers of CpG ODNs, which can significantly improve the delivery of CpG ODNs, as shown by the significantly enhanced cellular uptake and significantly increased levels of cytokines and nitric oxide produced following CpG ODN stimulation (Zhang et al., 2019).

SF has particularly excellent biocompatibility and can slowly degrade *in vivo*, has an excellent mechanical strength, and increases cell adhesion. Injectable hydrogels prepared by combining SF with other materials achieve controlled biodegradation and low mass loss and can be loaded with immunosuppressive agents such as methylprednisolone and betamethasone for cartilage regeneration and the treatment of rheumatoid arthritis (Phan et al., 2022; Oliveira et al., 2020).

Human bone marrow mesenchymal stem cells (hBMSC) are widely used in cell therapy because of their powerful proliferative and immune-regulatory abilities. SF films reportedly preserve not only the immunosuppressive effects of hBMSCs on T-cell proliferation and cytokine release, but also IL-6 secretion, and the immunophenotypes of hBMSCs (Luan et al., 2009).

### 5.2 Anti-inflammatory properties

#### 5.2.1 Inhibition of inflammation via promotion of M2 polarization of macrophages

M2 macrophage polarization is an important immune regulatory event that reduces inflammation during wound repair, bone regeneration and repair, and colitis repair. Many composite biomaterials targeting this key event have been developed based on the excellent mechanical properties, biocompatibility, and bioactivity of SF. For example, Silk-6/ε-PL@Exo (constructed from SF/poly-L-lysine hydrogel) controls inflammation, inhibits glycolysis and lactic acid accumulation by targeting M1 macrophages, and promotes the polarization of macrophages from M1 to M2 (Jin et al., 2024). The inflammatory response induced by SF is not fixed: SF/nano-hydroxyapatite scaffolds trigger a proinflammatory response by M1 macrophages on the first day, whereas SF degradation products induce an anti-inflammatory response by M2 macrophages on the day 24 of treatment (Wong et al., 2024). Lv et al. found that SF treated with reagents such as acetic acid and sodium hydroxide increased the expression of proinflammatory cytokines in rats and accelerated their degradation *in vivo*. Overall, most studies suggest that SF has good biocompatibility and anti-inflammatory properties.

Owing to the dissolution of the scaffold by protease K in the body, the secondary/tertiary structure of SF is altered, leading to significantly different immune responses. Maintaining the stability of SF in the body is of great clinical value, and bioactive gold cluster sutures (clusters assembled on the SF surface) ensure the structural stability of SF for 15 months without degradation *in vivo* (Tian et al., 2024).

##### 5.2.1.1 Wound treatment and macrophage polarization

**FIGURE 5:**
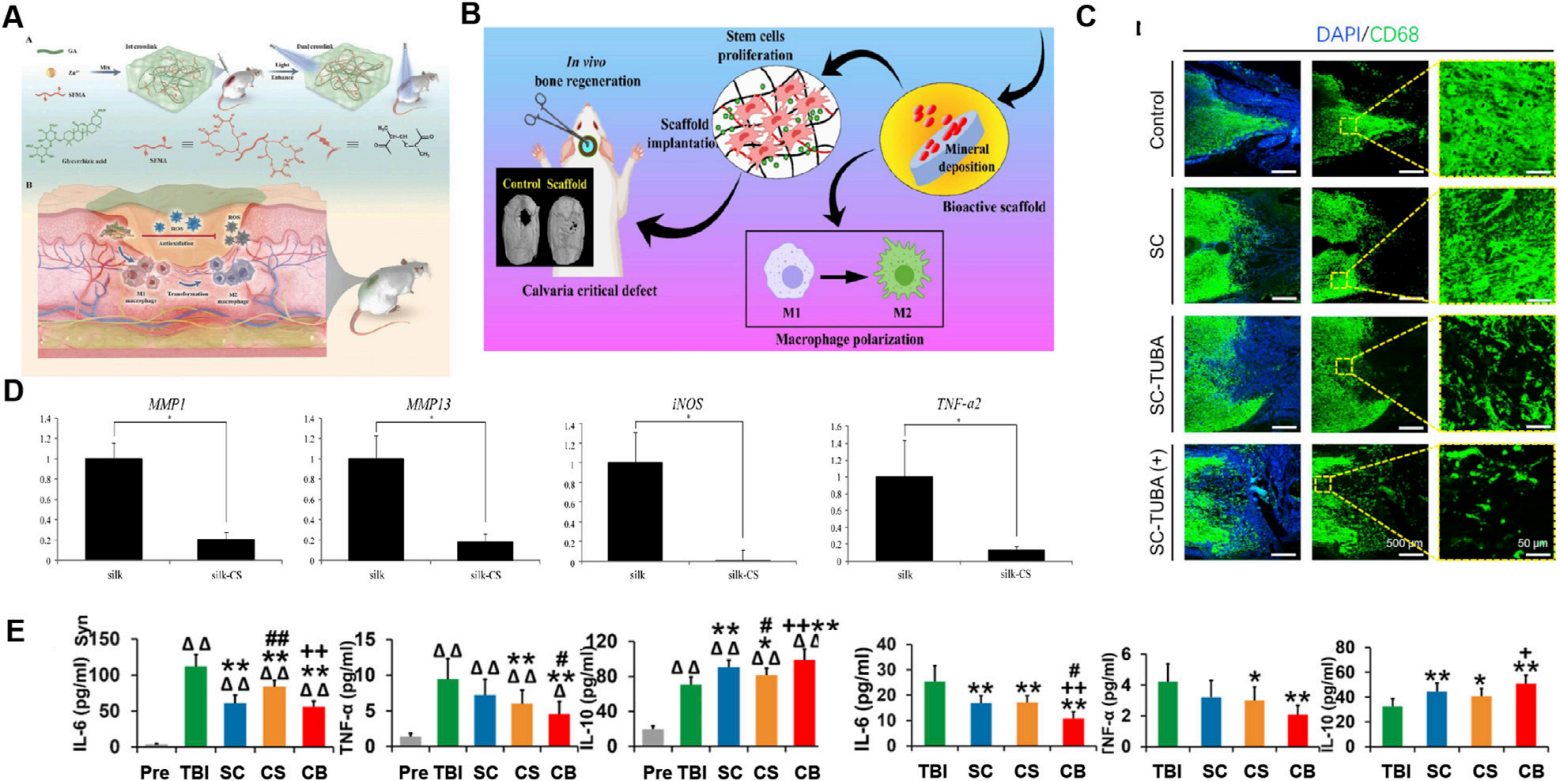
Application of SF-based biomedical composites in inhibiting inflammation. **(A)** Preparation of SF/GA/Zn hybrid hydrogel and its immunomodulatory mechanism in wound treatment of diabetes. Reprinted with permission from (Qian Y. et al., 2022). Copyright (2022) Wiley-VCH. **(B)** Schematic of the osteo-immunomodulatory effects of three-dimensional-printed biodegradable cellulose nanoparticles-reinforced chitosan/silk fibroin (CS/SF/CNPs) scaffolds. Reprinted with permission from (Patel et al., 2022). Copyright (2022) Elsevier. **(C)** The CD68 antibody fluorescence staining image confirmed the inhibitory effect of the hybrid poly (glycolide-co-ε-caprolactone) (PGCL)/SF-Tubasatin A (TUBA) multi-channel bioactive filament nanofiber catheters on inflammation after SCI. Reprinted with permission from (Liao et al., 2022). Copyright (2022) Elsevier. **(D)** RT-PCR displays the expression levels of genes *MMP-1, MMP-13, iNOS*, and *TNF-*α*2* activated by IL-1β, were dramatically lower in the silk-chondroitin sulfate (CS) scaffolds compared with the silk scaffold. Reprinted with permission from (Zhou et al., 2017). Copyright (2017) Elsevier. **(E)** The expression of IL-6, TNF-α, and IL-10 showed that the implantation of collagen/SF scaffold combined with human umbilical cord mesenchymal stem cells (hUCMSCs) regulated systemic inflammatory factor levels in the acute and chronic stages of traumatic brain injury. Reprinted with permission from (Jiang J. et al., 2021). Copyright (2021) Theranostics.

Wound treatment promotes M2 polarization of macrophages to establish anti-inflammatory niche required for tissue healing, which is critical for skin wound treatment. SF hydrogels can significantly increase the expression of the anti-inflammatory marker CD163 in M2 macrophages in the early stage, accelerating the transition from inflammation to the proliferation stage of wound repair (Chouhan et al., 2018). Considering that the SF hydrogel system has a controlled drug delivery capacity, good mechanical properties, skin tissue adhesion, and bioactivity, the combination of other biomaterials and therapeutic drugs will not only be beneficial for promoting angiogenesis in the wound area but will also induce M2 polarization in the wound area to create a pro-healing anti-inflammatory microenvironment. For example, after *in situ* photocuring, methacrylonyloxylated SF hydrogel showed good adhesion and sealing properties. Currently, two methacryloxylated SF hydrogel systems loaded with borosilicate and metformin have been prepared, both of which can regulate inflammation by inducing macrophage polarization towards the anti-inflammatory phenotype M2 and support diabetic wound treatment (Pang et al., 2021; Mei et al., 2022; Wu et al., 2021; Wang et al., 2023; Li et al., 2023). In addition, novel glycyrrhizic acid and inorganic zinc in the immunoregulatory SF/novel glycyrrhizic acid/inorganic Zn2^+^ (SF/GA/Zn) hydrogels synergistically reduce the activation of M1-type macrophages, induced an M2 phenotype shift, and accelerate the three stages of diabetic wound repair (Qian Y. et al., 2022) (Figure 5A).

SF can co-self-assemble with VEGF-mimicking peptides to construct an immunoregulatory hydrogel, QK-SF, which supports tissue repair and wound healing by regulating macrophage polarization and promoting angiogenesis (Chen Z. et al., 2023). Similarly, gelatin methacrylate/silk fibroin glycidyl methacrylate/mesoporous silica NP-resveratrol/platelet-derived extracellular vesicles (GelMA/SFMA/MSN-RES/PDEVs) hydrogels have appropriate mechanical properties and swelling ratios, which allow sustainable release of MS-RES and PDEVs to regulate macrophage to M2 phenotype conversion, promote angiogenesis, and accelerate the diabetic wound treatment process (Zhu et al., 2022).

##### 5.2.1.2 Bone regeneration and repair

During the process of fracture healing, the first stage is acute inflammation, followed by a transition to repair and regeneration. Therefore, the development of bone immunoregulatory biomaterials that favor polarization of the M2-phenotype macrophages is a novel strategy for bone regeneration and repair (Hu et al., 2018). SFNPs improve the bioavailability of hydrophobic anti-inflammatory drugs. Ti-MAO/Sr/LBL_WNP_ prepared according to this strategy can continuously release wogonin, which can transform M1 macrophages into M2 macrophages, regulate the ratio of M1/M2 macrophages, and promote osteoblast differentiation (Wang D. et al., 2022). SF also has the ability to release cytokines locally and can be used for the local release of sitagliptin to induce macrophages to polarize to the M2 phenotype and effectively recruit M2 macrophages to the titanium implant site to support bone regeneration (Xiang et al., 2021). In addition, the addition of SF/cellulose nanofibrils (CNFs) makes three-dimensional printing of chitosan/SF/cellulose nanoparticle scaffolds, and on activation of the M2 phenotype, macrophage polarization and immune regulation contribute to bone regeneration (Patel et al., 2022) (Figure 5B). A composite hydrogel composed of photoresponsive methacrylate SF, laponite nanocomposite, and tannic acid has the ability to resist oxidation and inflammation and induce bone formation (Wang et al., 2024). SF/black phosphorus/lycrhizic acid nanocomposite hydrogels can weaken the damage caused by oxygen free radicals, promote macrophage polarization towards M2, inhibit proinflammatory effects, and enhance the repair of damaged bone marrow (Zhang B. et al., 2024). Multilayered regenerated SF (RSF) on the surface of PET artificial ligaments regulates the inflammatory response and promotes the maturation of intra-articular grafts (Chen N. et al., 2023).

##### 5.2.1.3 Colitis

Oral nanoparticles have been used to treat ulcerative (UC) as they can deliver drugs directly to the colonic region and are more convenient, achieving high patient compliance and safety (Zu et al., 2021). Therapeutic strategies for the treatment of UC include enhancing inflammation resolution, alleviating oxidative stress, promoting colonic mucosal repair, and regulating the intestinal flora. Macrophage M2 polarization is conducive to resolving inflammation and promoting mucosal healing, and has recently been shown to involved in the treatment of inflammatory bowel disease (IBD) (Koelink et al., 2020; Sun et al., 2020). At present, improving the targeting and co-treatment of UC from multiple aspects is the most recent strategy for preparing UC therapeutic drug delivery systems. Specific ideas include (i) modifying oral nanoparticles with ligands that specifically target colonic epithelial cells or macrophages (Li et al., 2022) and (ii) using drugs with multiple therapeutic functions (Zu et al., 2021). In line with this strategy, SFNPs have been designed as oral nanodrug delivery systems to exert multiple therapeutic effects (such as promoting macrophage M2 polarization) by the targeted delivery of therapeutic agents to the colonic mucosa and to significantly alleviate UC symptoms (Liu et al., 2022; Du et al., 2022; Ma et al., 2022; Gou et al., 2019).

In addition, the prepared SF composite biomaterials with immune regulatory functions can regulate macrophage M2 polarization, which is used to promote tendon repair, prevent tendon adhesion, and promote pelvic floor tissue repair (Cai et al., 2023; Dong et al., 2021; Yin et al., 2022).

#### 5.2.2 Inhibition the infiltration and proliferation of inflammatory immune cells

Composite biomaterial systems in the form of hydrogels, scaffolds, and nanoparticles prepared using SF can be loaded with anti-inflammatory drugs, endowing them with anti-inflammatory activity by inhibiting the infiltration and proliferation of inflammatory immune cells. For example, SF hydrogels loaded with EGCG, rhein, and glycyrrhizic acid can effectively reduce the infiltration and proliferation of inflammatory cells (Qian Y. et al., 2022; Yin et al., 2022; Lee et al., 2022; Zhang F. et al., 2022). For the scaffold system, a gelatin sponge scaffold modified with neurotrophin-3 (NT-3)/SF can achieve a controlled-artificial release system with significant inflammatory inhibitory activity in the rat spinal cord injury (SCI) model, as shown by a significant reduction in the number of IBA-1 positive and CD68 positive macrophages/microglia (Li et al., 2018; Li G. et al., 2016). SF NP drug delivery systems loaded with bromelain and ZnO NPs, EGCG and Tubasatin A is effective in reducing the massive infiltration and proliferation of inflammatory cells (Liu et al., 2022; Hasannasab et al., 2021; Xie et al., 2022; Liao et al., 2022) (Figure 5C).

In particular, it is worth noting that SFNPs have anti-inflammatory properties, which can inhibit the infiltration and proliferation of inflammatory immune cells and can cooperate with anti-inflammatory drugs to exert anti-inflammatory effects. Therefore, SFNPs are a suitable choice for the preparation of anti-inflammatory composite biomaterials.

#### 5.2.3 Repair of immune homeostasis by modulating the release of inflammatory factors

##### 5.2.3.1 Wound treatment and induction of inflammatory factors

SF exhibits excellent biocompatibility, very low immunogenicity, great modification potential, and can regulate wound treatment process through the NF-κB signaling pathway, and thus, it has attracted much attention (Chouhan and Mandal, 2020). However, SF materials are brittle and have rapid enzymatic biodegradability, which limits their application in wound healing. Therefore, they are usually complemented with other polymers to optimize their effects for wound treatment (Shen et al., 2022). SF hydrogels, scaffolds, and nanofibrous membrane systems have been developed to promote wound treatment by loading anti-inflammatory drugs and regulating the release of inflammatory factors to repair immune homeostasis. The SF hydrogel system can reduce the expression of IL-1, IL-6, IL-8 and TNF-α, and can increase the expression of IL-10, TGF-β, and Arg-1 by loading resveratrol, novel glycyrrhiza, CNP-miR146a, and borosilicate (BS). Inhibition of inflammation in the wound microenvironment accelerates the transition from the inflammatory to proliferative phase, thereby accelerating diabetic wound treatment (Niemiec et al., 2020; Pang et al., 2021; Qian Y. et al., 2022; Zhu et al., 2022; Yang et al., 2024). In addition, SF composite biomaterials loaded with rhein and puerarin promote the inflammatory stage of the wound by reducing the levels of IL-6, Inos, COX-2 and TNF-α, and correspondingly increasing the levels of IL-10, thereby promoting wound treatment process (Liu et al., 2022; Yin et al., 2022).

##### 5.2.3.2 Articular cartilage repair

IL-1β is a proinflammatory factor, which induces inflammation and hinders articular cartilage repair (Wojdasiewicz et al., 2014). Therefore, it is an effective therapeutic strategy to use SF composite biomaterials system to deliver anti-inflammatory drugs to inhibit IL-1β and create an anti-inflammatory microenvironment to promote the repair of articular cartilage. At present, ginsenoside Rb1/TGF-β1-loaded biodegradable SF-gelatin scaffolds, SF-chondroitin sulfate scaffolds and injectable SF hydrogels containing articular chondrocytes (ACs) and hypoxic preconditioned exosomes (H-Exos) (SF/ACs/H-Exos) have been developed, all of which can reduce the inflammatory response of chondrocytes induced by IL-1β and support cartilage regeneration (Shen et al., 2022; Zhou et al., 2017; Wu et al., 2020) (Figure 5D).

##### 5.2.3.3 Colitis and delivery of anti-inflammatory drugs

As a non-toxic drug carrier with good biocompatibility, immunogenicity, and low biodegradability, SF can effectively treat colitis by preparing a NP system to deliver anti-inflammatory drugs to the inflamed parts of the colon. It is reported that SFNPs system loaded with pluronic F127 (PF127) -modified resveratrol (RSV), EGCG, patchouli alcohol (PA), and curcumin (CUR) can downregulate proinflammatory cytokines such as IL-1β, IL-6, IL-12, and TNF-α, upregulate anti-inflammatory cytokines such as IL-10, thus effectively alleviating the inflammatory response (Liu et al., 2022; Du et al., 2022; Gou et al., 2019; Xie et al., 2022).

##### 5.2.3.4 Nerve regeneration

Stem cell transplantation and biological scaffold implantation are considered effective methods for nerve regeneration. Safety and biocompatibility are two key factors in material selection for nerve regeneration research. SF is an excellent carrier for cell and growth factor delivery, a natural material with good biocompatibility, good mechanical properties, and biodegradability, and is reportedly a favorable choice for the repair of SCI and traumatic brain injury (Xu et al., 2016; Jiang et al., 2020). The inflammatory response in the injured area of the nervous system will inhibit nerve regeneration; thus, SF scaffolds with anti-inflammatory effects have also been developed, mainly by reducing the proinflammatory cytokines IL-6 and TNF-α, and increasing the anti-inflammatory cytokines IL-10 to inhibit inflammation and promote nerve regeneration (Li G. et al., 2016; Jiang J. et al., 2021) (Figure 5E).

SF hydrogel significantly improves skin penetration and the anti-keratinization ability of curcumin-loaded NPs (CUR-NPs), and can prolong the release of curcumin-loaded NPs; thus, inhibition of inflammatory cytokines (TNF-αand IL-6) is achieved to a greater extent with improvements in the therapeutic efficacy of curcumin on psoriasis mouse model (Mao et al., 2017). In addition, neutrophil membrane-coated SF-NPs can enhance the bioavailability and solubility of ferulic acid (FA), improve its pharmacological characteristics and targeted delivery, thereby significantly reducing proinflammatory cytokines IL-6, IL-1β and TNF-α (Hassanzadeh et al., 2021). Similarly, liquid SF along with the RES-SFN treatment achieved better results than each of these treatments used separately, and showed a more significant reduction in the proinflammatory cytokines IL-1β and TGF-β (Giménez-Siurana et al., 2020).

## 6 Conclusion

SF and sericin have been widely used in wound treatment, tissue engineering, and other fields in the form of hydrogels, scaffolds, films, and nanoparticles in recent decades. These different processing methods enable them to play different roles in the wound treatment process (proinflammatory and antibacterial stage or anti-inflammatory and healing promoting stage).

**FIGURE 6:**
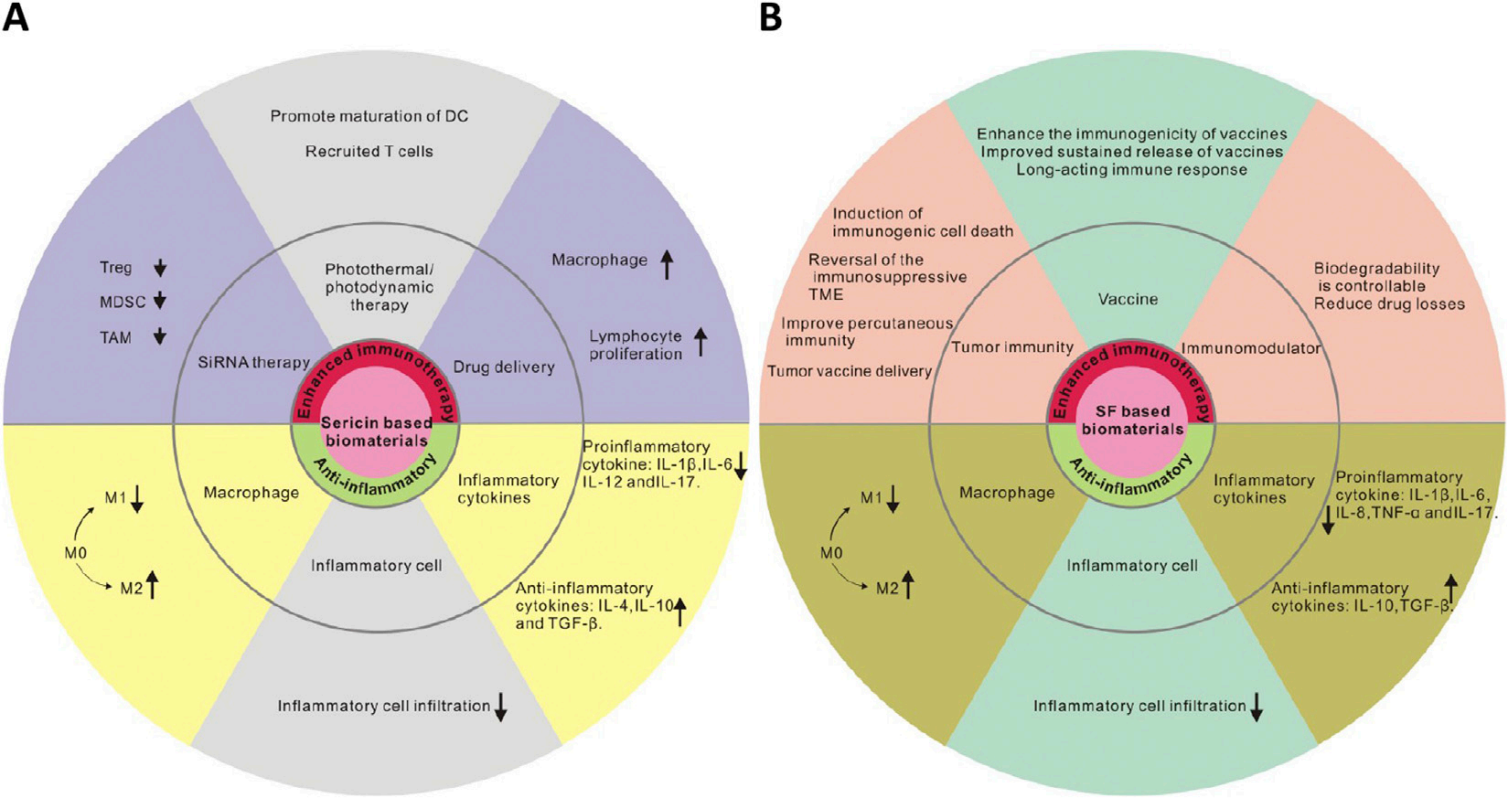
Immune response induced by **(A)** sericin/**(B)** SF-based biomaterials *in vivo* and their application.

Our review focused on the anti-inflammatory and immunoregulatory effects of SF and sericin as biomaterials, particularly in the field of wound treatment. SF and sericin have been shown to be safe, biocompatible, and to exhibit low immunogenicity, and can elicit appropriate and acceptable immune responses *in vivo*, including innate and adaptive immune responses, when used alone or prepared in various forms of biomaterials. The anti-inflammatory and immune regulatory properties of sericin and SF make them widely used in skin wound treatment, UC, articular cartilage repair, and psoriasis. In addition to individual applications, they can also be combined with other materials to make composite materials using their modifiability, controllable biodegradability, and good mechanical properties, which can not only be used to improve the properties of single anti-inflammatory drugs, but also give full play to their own immune regulation and anti-inflammatory ability. These composites can improve existing immunotherapy methods, such as delivery of siRNA, enhancement of cellular immunity, and can be exploited as vaccine carriers to exert immune regulation. SF and sericin composites can also exert anti-inflammatory effects by promoting M2 polarization of macrophages, inhibiting the proliferation and infiltration of inflammatory cells, and regulating the release of inflammatory factors (Figure 6).

There are still many challenges to be faced regarding the application of sericin-based and SF-based biomaterials in the field of medical biomedicine. For instance, due to the lack of standardized methods for assessing immune responses, it is difficult to make a comprehensive assessment of implant-induced immune responses (Kaprin et al., 2022). Additionally, it has also been well-known that epithelial cells and other types of immune cells (such as mast cells) equally play a significant role in the direct immune responses. However, there are few researches have explored their inflammatory response to sericin-based and SF-based biomaterials. Last but not least, there are some limitations of sericin-based and SF-based biomaterials in wound applications: (i) The extraction and purification process of SF and sericin is complicated, and other substances are easy to remain, which affects the purity and properties of the biomaterials; (ii) Compared to sericin, SF lacks antibacterial and antioxidant properties, so it is not effective in preventing wound infection.

In conclusion, SF and sericin exhibit good biocompatibility, low immunogenicity, controllable biodegradability, good mechanical properties, and excellent anti-inflammatory and immunomodulatory properties. A profound understanding of the different ways in which SF/sericin acts as a biomaterial and induces either proinflammatory or hypoinflammatory responses in the body will greatly improve utilization rate of silk biomaterials, especially in the field of wound treatment.

## Abbreviations

CH, chitosan; SF, silk fibroin; SS1, sericin; HBsAg, hepatitis B surface antigen; IL-1/5/8/12/16/23, interleukin-1/5/8/12/16/23; TNF-α, tumor necrosis factor alpha; iNOS, inducible nitric oxide synthase; COX-2, Cyclooxygenase-2; LMW, low molecular weight; MMP, metalloproteinases; M1, classical activated macrophages; M2, Alternatively activated macrophages; BMP, morphogenetic protein; UC, ulcerative colitis; RA, rheumatoid arthritis; NF-κB, nuclear factor kappa-B; MAPK, mitogen-activated protein kinase; BM, *Bombyx mori*; PEG, polyethylene glycol; SCI, spinal cord injury.
